# Enhancing pre-school teachers’ competence in managing pediatric injuries in Pemba Island, Zanzibar

**DOI:** 10.1186/s12887-022-03765-6

**Published:** 2022-12-02

**Authors:** Mohamed A. Salim, Prosper Gabrieli, Walter C. Millanzi

**Affiliations:** 1 Department of Nursing Management and Education, School of Nursing and Public Health, University of Dodoma, Dodoma, Tanzania; 2 Department of Educational Psychology and Curriculum Studies, College of Education, University of Dodoma, Dodoma, Tanzania

**Keywords:** Pre-school teachers, Pediatric injuries, First aid, Pre-school children, Pemba Island

## Abstract

**Background:**

Safe and healthy learning environment in pre-schools has received increased attention in promoting the well-being of pre-school children. However, pediatric injuries have remained one of the leading causes of childhood morbidity and mortality around the globe. Empowering pre-school teachers with first aid competencies have been identified as being of potential strategy against pediatric health burdens of problem. This study tested the effect of established pediatric first aid training on pre-school teachers’ knowledge, attitude, and intention to practice first aid management to pre-school children in Zanzibar.

**Methods:**

Uncontrolled quasi-experimental design with a quantitative research approach was conducted in Teachers’ Resource Centers among 120 preschool teachers at Pemba Island, Zanzibar. First aid training was facilitated based on the prescribed guidelines and standards of materials adapted from the American Academy of Pediatrics and implemented. The intervention was preceded by a baseline assessment using structured questionnaires adopted from previous studies that served as the main data collection tool.

**Results:**

Participants’ mean age was 32 years ± 6.2 with 84.2% of the sample being females. Given the training, post-test findings demonstrated a significant increase (*p* < 0.01) in participants’ first aid management scores with mean differences of M = 15.08 ± 5.34 (Knowledge), M = 26.99 ± 6.587 (Attitude), and (M = 4.76 ± 0.648 (Intentional practice).

**Conclusion:**

The established pediatric first aid training can enhance the spectrum of managing pediatric injuries among preschool teachers in Zanzibar. Ongoing public health services opportunities should be structured within teachers’ continuous learning against pediatric injuries in Zanzibar-Tanzania.

## Introduction

A safe and healthy learning environment in pre-schools has received increased attention in promoting the well-being of pre-school children [1]. However, pediatric injuries have remained one of the leading causes of childhood morbidity and mortality around the globe. School children are the major risk group that faces traumatic conditions, like fractures, fainting, falling, drowning, and road traffic accidents which threaten their health during school life [2]. Recent studies indicate that pediatric injuries account for 10 to 25% of injuries worldwide, which are happening among children when at schools [3, 4]. Compared with adults, more than six million children and young adults were injured seriously and needed emergency hospital services [5, 6].

Available statistics show that 300 children and teenagers die in Africa, and most of them are dying from unintentional injuries, drowning, poisoning falls, burns, and violence [7]. Research shows that unintentional injuries among children usually happen frequently on school premises, especially on the sports ground [8]. In Tanzania for example, 2.5% and 4.3% of persons were reported to have been injured during the previous year in urban and rural areas respectively of which 37% of the injured people were children below 14 years. An epidemiological study that was conducted in 2012 in rural and urban areas of Tanzania mainland demonstrates that 2.5% and 4.3% of individuals were wounded in the urban and rural areas respectively [9, 10].

Among the injuries, 37% were children below 14 years. Teenagers below 12 years have a greater vulnerability to injuries due to frequent falls [11]. A cross-sectional survey conducted in Tanzania revealed that 47% of under-five children are at great risk of experiencing pediatric injuries [3]. Incidences of pediatric injuries in some districts of Pemba Island in Zanzibar increases tremendously from a reported 4371 cases in 2018 to 4998 cases in 2019. Data indicate that injury cases in WETE and MICHEWENI districts are relatively higher. In terms of numbers, the cases are relatively lower in CHAKECHAKE and MKOANI districts. In some developing regions such as Zanzibar-Tanzania, lack of health workers (nurses and or doctors) in schools, and the distance from schools to health centers is relatively long for timely accessibility of emergency health services once a child is injured in school [12].

Despite these challenges, the ministry of health in Zanzibar, through its five-year development plan (FYDP), objective number four, targets to improve partnerships among the public–private sector, private, sector, religious institutions, civil society organizations, and the community in the provision of health services. Experiences indicate that all these interventions focus only on the health centers alongside their health personnel and hardly involve other personnel such as teachers who are seemingly closest to the pupils in schools [13]. With this regard, schools are very important ideal locations to consider when focusing on the prevention of injuries associated with pediatric conflicts, playing activities, the presence of swimming pools in some schools, and/or nutrition-related health emergencies such as hypoglycemia [14, 15].

However, scholarly works [16, 17] demonstrate that teachers in pre-schools have not yet been exposed to any formal training to provide first aid services to injured children. Yet, no single course in any pre-service program offers pre-school teachers opportunities to learn how to provide first aid service to pre-school children. In this situation, teachers feel not concerned and hold little support when confronting pediatric injuries and thus, may have the perception that it is the role of healthcare personnel [18]. Now, it seems important to provide pre-school teachers with an opportunity to enhance their knowledge, attitude, and intentions to provide first aid services to daycare and boarding preschools.

Attempting to enhance teachers’ knowledge, attitude, and competencies in providing first aid services are not new in the world. The American Academy of Pediatrics (AAP) brought a pediatric first aid course and first aid training among caregivers and teachers in 2005 that focused on educating and empowering them to demonstrate the confidence they need to care for sick and or injured children effectively [19]. As it has worked elsewhere, findings of the first aid training have revealed a positive influence on preschool teachers' and caregivers’ competencies in managing pediatric emergencies in schools and homes respectively [20–23].

However, little has been unfolded on the sustainable multidisciplinary pedagogical strategies to address them among children in pre-schools. an attempt to involve preschool teachers’ interventional programs to learn the provision of pediatric first aid has not yet been established in Zanzibar, Pemba Island in particular. In this paper, we are reporting findings from the first aid training among pre-school teachers that aimed at enhancing their knowledge, attitude, and intention to provide first aid to pre-school children in Pemba Island.

## Methods and materials

### Study design and approach

This study aimed at enhancing preschool teachers’ competence in managing pediatric injuries in preschool children in Pemba Island, Zanzibar from September to April 2020. The study adopted an uncontrolled quasi-experimental design that consisted of a pre-test that established the preschool teachers’ baseline knowledge, attitude, and intention to practice first aid management at school premises. It was implemented in teachers’ resource centers at Zanzibar based on the institutional regulations and guidelines for postgraduate studies at the University of Dodoma, Tanzania. Quasi-experimental studies can be implemented quantitatively, qualitatively, or in a mixed research approach either in a controlled (Having two groups of which one serves as intervention/treatment and another as a control group respectively) and uncontrolled style (Having only one group that is treated as the intervention and control) [24].

The post-test served as an end-line assessment to measure the effect of the intervention on preschool teachers’ knowledge, attitude, and intention to practice first aid management to preschool children at schools. The post-test questionnaires had an equivalent number of items per variable to the pre-test questionnaires that were administered to the same participants during baseline assessment. The consented participants filled out the pre-and-post-intervention questionnaires in a separate unoccupied class to ensure confidentiality and privacy. Brief instructions on how to fill out questionnaires were provided by the principal investigator and assistants before distributing them to study participants.

The principal investigator and the assistant supervised the process and were available to respond to participants’ queries throughout the process. A schedule for the training was then shared among the study participants, which was scheduled to start a week later after the day of the pre-test (baseline assessment). Using the same study site, the design was aligned with a quantitative research approach to quantify preschool teachers’ sociodemographic characteristics profiles and variables of interests under study including the intervention (independent variable), first aid knowledge, attitude, and intention to practice the provision of pediatric first aid services to pre-school children. The design was opted to cater to the simplicity of quantifying the variables under study and controlling information contamination among the consented preschool teachers.

### The intervention

#### Training materials

To assure the validity of the training, first aid training materials used in this study were adapted from the American Academy of Pediatrics and implemented based on the prescribed guidelines and standards [19].

#### Intervention implementation team

Information in Table 1 shows that 15-trained personnel who also had expertise in teaching and or providing health services implemented the training process as a study intervention. They included nine males and six females based on their consent to take the assigned roles of the intervention. The trained personnel were required to have at least a tertiary level of education with working experience of at least 1 year to be recruited in the study. Out of 15 trained personnel, 4 (26.7%) were nurse educators, 3 (20.0%) were clinical instructors, 3 (20.0%) were medical doctors and 5 (33.3%) were teachers. All of the trained personnel were residing in Zanzibar. Each trained personnel was assigned to train one group of study participants throughout the intervention timelines.

**Table 1:** Sociodemographic characteristics profiles of trained personnel for the Intervention (*n* = 15)

Variable	n(%)
**Sex**	
Male	9(60.0)
Female	6(40.0)
**Residence**	
Micheweni	4(26.7)
Wete	6(40.0)
ChakeChake	5(33.3)
**Professional Education**	
Certificate	3(20.0)
Diploma	6(40.0)
Bachelor	5(33.3)
Master	1(6.7)
**Professional qualification**	
Teacher	5(33.3)
Nurse Educator	4(26.7)
Clinical Nurse	3(20.0)
Medical Doctor	3(20.0)
**Working experience**	
1 year	2(13.3)
2 – 5 years	9(60.0)
> 5 years	4(26.7)

#### Timelines of the intervention to the administration of post-tests among study participants

The intervention was conducted in Zanzibar using unoccupied Teachers’ Resource Centers (TRCs) as venues for the first aid management training sessions. As shown in Table 2, the intervention consisted of four phases including phase one (September to January 2020) which consisted of the development of a proposal and first aid training materials, experts’ appraisal, and prototyping of the first aid training materials. Phase two (February 2020) involved the recruitment of the study participants and baseline assessment to record pre-school teachers’ sociodemographic characteristics profiles, prior knowledge, attitude, and intention to provide pediatric first aid services to pre-school children. Phase three (March to April 2020) was six weeks of first aid training among pre-school teachers with a duration of three sessions per day. Phase four (April 2020) served as an end-line assessment in which study participants were administered post-tests to assess their first aid knowledge, attitude, and intention to practice first aid management to preschool children on preschool premises. Post-test (End-line assessment) was set to be administered immediately after the intervention, which was defined in this study as one week after first aid management training.

**Table 2:** Summary of the study timeline of the intervention to the administration of post-tests among study participants

Activities	Phases			
	**I (September to January)**	**II (February 2020)**	**III (March to April 2020)**	**IV (April 2020)**
Development of proposal and First aid training materials	✓	×	×	×
Experts’ appraisal of the first aid training materials	✓	×	×	×
Prototyping of the first aid training materials	✓	×	×	×
Recruitment of the study participants	×	✓	×	×
Baseline assessment (pre-test)	×	✓	×	×
Training of the study participants using the appraised first aid training materials	×	×	✓	×
End-line assessment (Post-test)	×	×	×	✓

*Key*

✓ = the activities was performed

** × ** = the activity was not performed

#### Composition of the training package

The training was implemented in groups of which each group consisted of eight members making a total of 15 groups that were trained for 6 weeks with an average of one topic per session based on the negotiated schedule of the sampled schools. As indicated in Table 3, brainstorming, discussions, 5 to 8 members’ group works, video, and demonstrations were the main pedagogical strategies used to facilitate first-aid learning among preschool teachers. Sessions’ duration ranged from 30 to 90 min. Evening hours (From 03:00 pm to approximately 04:00 pm) were used to facilitate the training not only as negotiated by the study participants but, also as a mechanism of not interrupting schools' teaching and learning activities. Trained personnel (nurse educators, clinical instructors, and medical doctors) would have finished their morning duties at their working stations and have some rest to implement the intervention.

**Table 3:** Summary of the pediatric first aid training program

	***Week I***		***Week IV***	
30 min	**Session 1:** Overview of first aid and first aid Kit *Method:* Brainstorming, group works, Discussions and walking through the guide	90 min	**Session 4:** First aid management of a child with fainting *Method:* Brainstorming, group works, Discussions and walking through the guide	Researchers
30 min	Overview of the first aid resources/kits *Method:* Brainstorming, group works, Discussion and working through the guide			
	***Week II***		***Week V***	
90 min	**Session 2:** First aid management of a child with a fracture *Method:* Video, Demonstrations and practices	90 min	**Session 5:** First aid management of the child with open and punctured wound *Method:* Video, Demonstrations and practices	Researchers
	***Week III***		***Week VI***	
90 min	**Session 3:** First aid management of a child with Hypoglycemia/Fainting *Method:* Video, Demonstrations and practices	90 min	**Session 6:** First aid management of the child who sustained drowning *Method:* Video, Demonstrations and practices	Researchers
30 min	**Week VII:**Administration of Post-test			Researchers

The training involved the theoretical part (Concepts of first aid including its characteristics, principles, advantages, indications/health problems that may need first aid, resources/first aid kit needed for first aid) and the practical part (Facilitators’ demonstrations and return demonstrations by the study participants on when and how to provide first aid management to children). The first two weeks of the training were used for facilitating the theoretical part of the package whereas the other four weeks were for practical activities. Demonstrations were flexible based on participants’ requests to repeat them to the saturation, which also served as a formative assessment of their mastery in providing first aid management to pre-school children.

### Participants and settings

A stratified random sampling technique by random numbers table was used to select 45 government-based schools out of 55 and 30 private-based schools out of 37 located in the north Pemba region in Zanzibar islands, Tanzania. The type of school based on ownership was set as a criterion to stratify schools into two strata including government-owned schools and private-owned schools strata respectively. A statistician independent of this study performed stratified sampling procedures. The consented preschool teachers working in the selected study settings were included in the study with an exception of those who were sick, participating in other projects, and those who were under school special activities.

#### Sample size determination

**Fig. 1:**
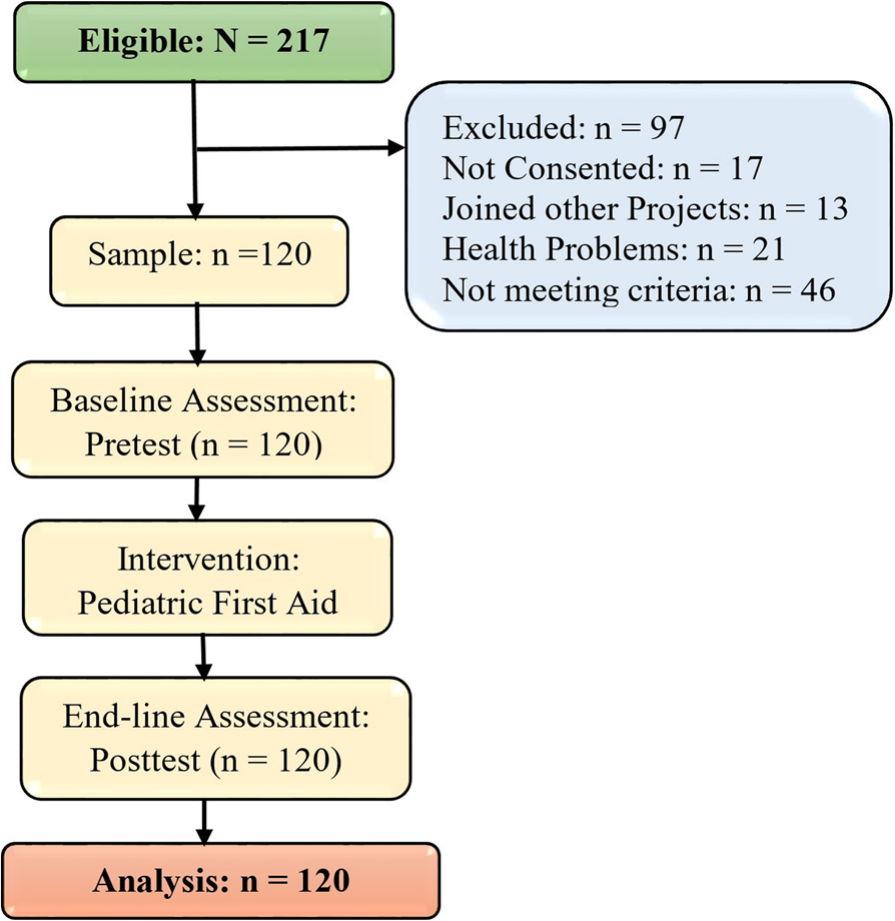
Flow pattern showing the screened, recriuited, intervened and analysed study participants

A total of 217 preschool teachers were eligible to join the study. The following formula was used to calculate the minimum sample size of the current study as recommended by previous studies

1 $$n=\frac{{\left\{\mathrm{\rm Z}\mathrm{\alpha }\surd [\mathrm{\pi o }(1-\mathrm{\pi o})] +\mathrm{Z\beta }\surd [\uppi 1 (1-\uppi 1)]\right\}}^{2}}{{(\uppi 1-\mathrm{\pi o})}^{2}}$$

whereas Zα was set at 1.96 from the normal distribution table; Zβ was set at 0.80; Mean zero (π_0_) and Mean one (π_1_) were adapted from the previous study. As shown in Fig. 1, 120 pre-school teachers were sampled to participate in the pediatric first aid training program conducted in two teachers’ resource centers (TRCs) and their information was analysed after the end-line.

### Data collection tools

Tables 4, 5, and 6 shows questionnaire items per outcome variable. The study adopted questionnaires from previous studies [4, 23, 25, 26] and modified by the principal investigator supported by the consulted research experts, colleagues and statisticians to fit the Tanzanian context.

**Table 4:** Questionnaires items for knowledge of respondents about first aid services

S/N	Item	Yes	No
1	Aim of the first aid is to save life?		
2	Air way, breathing and circulation are the priorities of first aid?		
3	Bandage, plaster and scissor are the main items in the first aid kit?		
4	We can check circulation by touching Adam's apple?		
5	CPR is a procedure to help a person who his/her heart beats and breaths stops?		
6	A child with an electrical shock needs Perform Cardiopulmonary Resuscitation (CPR)		
7	30 chest compressions and 2 rescue breaths is among CPR procedure		
8	To open air way is the first aid procedure for unconscious child		
9	Fracture is the break or creak of bone		
10	Open fracture is the fracture in which bone breaks the skin		
11	Pain and deformity are among the signs and symptoms of fracture		
12	Splinting is the first aid care for a child with a fracture		
13	Drowning is asphyxiation due to immersion in water		
14	To restore adequate breathing is the aim of first aid for drowning		
15	Abdominal thrush maneuver is to remove water from the abdomen of a child		
16	A wound is any type of injury to the skin		
17	A bandage is used to secure dressing to the bleeding wound		
18	Icepack is used to slow down bleeding and reduce swelling		
19	Low blood sugar is the one of the causes of fainting		
20	Loosing tight clothes is the first aid for fainting		

Instructions: The following items explores you First Aid comprehension. Please, put a tick (√) in an appropriate answer (only one tick per item)

**Table 5:** Questionnaires items for teachers’ attitudes towards first aid services

S/No	Question statement	Strongly agree	Agree	Strongly disagree	disagree
1	Giving first aid for children at school is fair	4	3	2	1
2	To provide first aid services at school is pleasant	4	3	2	1
3	To provide the first aid services to pre-school children is the duty of the teachers	4	3	2	1
4	It is very important for me to learn first aid	4	3	2	1
5	It is appropriate for teachers to provide first aid care to injured children at school	4	3	2	1
6	I have willingness to provide first aid care to children presented with any emergency situation at school	4	3	2	1
7	I believe that I have ability to provide appropriate first aid care to children at school	4	3	2	1
8	It is necessary to provide immediate first aid care to the children presented with any emergency condition in school	4	3	2	1
9	Keeping a first aid kit and special room is an obligation of school management	4	3	2	1
10	Any teacher is responsible to provide first aid services at school	4	3	2	1

Instructions: The following items explores your feelings towards First Aid services provision to children please put a tick (√) for an appropriate option in the box provided

**Table 6:** Questionnaires Items for Practice of respondents about the provision of first aid services to children

S/N	Item	Responses			
		**a**	**b**	**c**	**d**
1	What will you prefer to do first if you will have encountered a situation requiring first aid in school?	Administer first aid before seeking medical assistance first	Call a doctor first	Notify his/parents first	No sure about decision
2	What will you do if a child has a fracture of bone in the left arm?	Moving the child to home	Splinting the fractured arm then transfer to hospital	Call an ambulance driver	Notify his/parents
3	What will you do if a child encountered fainting?	Shout for help	Elevate the legs above the level of the head	Give water even a child is unconsciousness	No sure about decision
4	What will you do if you found a child is drowning (immersed) in water?	Run to call my fellow teachers	Remove the child from water but only if it is safe to do so	Shouting for help	No sure about decision
5	What will you do if you find child’s heart stop beating and breaths stop?	Perform Cardiopulmonary Resuscitation (CPR) with 30 chest compressions and 2 rescue breaths	Moving the child from class room to the office	Refer the child to hospital	No sure of the decision

Instructions: The following questions aim at understanding your day-to-day practices of providing first aid services to children. Please circle the appropriate answer to the letters provided (only one)

#### Characterization of a research tool

The tool collected participants’ socio-demographic data such as name, sex, residence, marital status, educational level, years of teaching experiences, areas of residence (10 items), knowledge (20 items) attitude (9 items), and intention to practice first aid management among pre-school children (5 items). For analysis purposes, the items' responses were structured into “Yes/No” with “Yes” responses valuing a “1” score and “No” having a value of “0” score. The endpoints of the outcome variables of interest were dichotomized for easy interpretations. Pediatric first aid knowledge was assessed on participants’ knowledge assessment basis while the intention to practice pediatric first aid management was as assessed in self-reported readiness and willingness to provide pediatric first aid services once they confront pediatric injuries among preschool children at school premises. Highest post-test scores in preschool teachers’ pediatric first aid knowledge (≥ 10 pass points), attitude (≥ 5 pass points), and intentions (≥ 3 pass points of any intended action that implied preschool teacher’s readiness and willingness to provide pediatric first aid service to preschool children) controlled with other factors at 95% confidence interval and probability α =  ≤ 5%(0.05) against the baseline findings was considered as a significant gain due to the intervention.

### Validity and reliability of the study

The research tools weres then shared with research experts, expert colleagues, and statisticians for their proof before subjecting them to a pilot study that was conducted among 20 pre-school teachers (10%) of the study sample before data collection. Exploratory factor analysis was performed for item reduction to get the highly weighed items above the statistically suggested threshold (> 0.3) as recommended by previous studies [27, 28]. The correlation coefficient was set at a cut-off point of ≥ 0.30 whereas, the Kaiser-Meyer-Oklin (KMO) value of ≥ 0.5 and probability α < 0.05 were used to assess sampling adequacy set at a cut-off point of ≥ 0.60. Findings of the explorative analysis of the questionnaires indicated that 34 items (Knowledge: *n* = 20 items; Attitude: *n* = 9 items and intention to practice first aid management: *n* = 5 items) weighed above a cut-off point of ≥ 0.30 and thus, were retained for further analysis.

Then a scale analysis was performed and findings revealed a Cronbach’s Alpha of 0.711 that bared approximately similar psychometric properties to the original questionnaires (α = 0.799). Findings from the reliability test implied a tool was reliable to be used in the actual field data collection and measure first aid knowledge, attitude, and intention to practice among pre-school teachers [29].

### Data analysis

The Statistical Packages for Social Sciences (SPSS) version 25 was used to analyze data. The tool was used for analysis to establish quantifiable data about preschool teachers’ knowledge, attitude, and intention to practice first aid management among preschool children, which were the objectives of this study respectively. Moreover, the statistical tool (SPSS) was adopted to establish the effect of an intervention over the outcome variables controlled for other factors such as pre-school teachers’ socio-demographic characteristics profiles. Descriptive analysis was performed to establish participants’ socio-demographic characteristics profiles. Mean score differences in the pediatric first aid knowledge, attitude, and intention to practice pediatric first aid management between the pre-test and post-tests were determined by paired t-test analysis model. As recommended by previous scholars, the effect size of the intervention over the outcome variables of interest was calculated using the effect size calculator for the t-test of the pre-posttest by Cohen’s *d* and then dividing the results by the pooled standard deviation [30, 31]. The following formula was used for calculating the effect size of the intervention in this study

2 $$Cohen\mathrm{^{\prime}}s d = (M2-M1)/{SD}_{pooled}$$

3 $$SD pooled = \surd (( {SD}_{1}^{2}+ {SD}_{2}^{2})/2$$

whereby $$M_2=Meantwo;M_1=Meanone;SD=Standard\;deviations\;and\;{SD}_{pooled}=Pooled\;standard\;deviations$$  

Univariate and multivariable logistic regression was performed to demonstrate the association between first aid training controlled to participants’ sociodemographic characteristics profiles and outcome variables (First aid knowledge, attitude, and intention to practice the provision of first aid services to preschool children). The confidence interval (CI) was set at 95%with the value of demonstrating a statistically significant difference set at α = 5% (*p* < 0.05) and β = 0.80 (Power of the study) to demonstrate the effect of the intervention controlled with other co-related factors over the outcome of interest under study. The effect size of ≥ 1 is equivalent to ≥ 10% of the gain in preschool teachers’ pediatric first aid knowledge, attitude, and intentions to provide pediatric first aid services to preschool children on school premises.

### Ethical consideration

The University of Dodoma (UDOM) Institutional Research Review Committee (IRRC) reviewed, approved, and issued an ethical permit for the study to be conducted through ethical clearance number UDOM/DRP/134/VOL VII/. The government of Zanzibar granted research ethical permit number OMPR/M.95/C.6/2/VOL. 6/13 and Zanzibar Health Research Institute (ZAHRI) through Zanzibar Health Research Ethical Committee (ZAHREC) granted research ethical permit number ZAHREC/03/ST/JUNE/2020/109 to reach and conduct the study in schools. Ethical Clearance to reach preschools was approved by the headteachers of the respective schools. Written informed consent was obtained from each participant by the principal investigator as one of the criteria for them to join the study. Anonymity procedures were adhered to ensure the confidentiality of participants’ particulars. Data were handled and secured by the principal investigator through a keyed folder.

## Results

The study recruited 120 pre-school teachers who completed all the study cycles (100% response rate). As shown in Table 7, the mean age of the study participants was 32 ± 6.2 years with 84.2% of the sample being females. The majority (78%) of them were working in the public sector.

**Table 7:** Pre-school teachers’ scio-demographic characteristics profile (*n* = 120)

*Variable*	*n (%)*
***Age of the pre-school teachers:*** (32.4 ± 6.2)	
18–35 years	84(70.0)
36–55 years	36(30.0)
***Gender***	
Female	101(84.2)
Male	19(15.8)
***Area of residence***	
Urban	73(60.8)
Rural	47(39.2)
***Sector of work***	
Public	94(78.3)
Private	26(21.7)
***Educational status***	
Secondary education	43(35.8)
Certificate in education	60(50.0)
Diploma in education	17(14.2)
***Working experiences***	
Short term experience	63(52.5)
Long term experiences	57(47.5)

### Mean score differences of participants’ knowledge, attitude, and intentions to practice first-aid management to children between pre-test and post-test

Findings in Table 8 illustrate the results from the paired t-test analysis model, which indicated that there was a statistically significant increase (*p* < 0.01) in participants’ first aid management scores after the training with mean differences of M = 7.47 ± 2.70 (Pre-test) and *M* = 15.08 ± 5.34 (Post-test) at t = 22.860 (t-value) set at a degree of freedom (df: n = 119). With regard to Cohen’s *d* classifications of effect sizes [32], the effect size of first aid management training on pre-school teachers’ knowledge was significantly high (Cohen’s *d* = 1.80). On the other hand, post-test findings demonstrated a significant gain (*p* < 0.01) in participants’ first aid management scores after the training with a mean difference of *M* = 11.45 ± 3.067 (Pre-test) and *M* = 26.99 ± 6.587 (Post-test) at 27.372 (t-value) set at a degree of freedom (df: *n* = 119).

**Table 8:** Mean score differences of participants’ knowledge, attitude and intentions to practice first-aid management to children between pre-test and post-test (*N* = 120)

*Variable*	*n*	*Mean (SD)*	*Mean diff*	*95% CI*		*t*	*p*	*Effect Size (Cohen’s d* = *(M*_*2*_* – M*_*1*_*)/SD *_*pooled*_*)*
				***Low***	***Upper***			
***Knowledge***								
Pre-test	120	7.47 ± 2.703	5.550	5.069	6.031	22.860	0.001	1.80
Post-test	120	15.08 ± 5.341						
***Attitude***								
Pre-test	120	11.45 ± 3.067	15.542	14.417	16.666	27.372	0.001	3.02
Post-test	120	26.99 ± 6.587						
***Intention***								
Pre-test	120	1.92 ± 1.553	2.842	1.655	3.145	18.808	0.001	2.39
Post-test	120	4.76 ± 0.648						

Based on Cohen’s *d* classifications of effect sizes, the effect size of first aid management training on pre-school teachers’ attitudes was significantly high (Cohen’s *d* = 3.02). Moreover, there was a significant increase (*p* < 0.01) in participants’ first aid management scores of intention to practice first aid management to preschool children after the training with mean differences of *M* = 1.92 ± 1.553 (Pre-test) and *M* = 4.76 ± 0.648 (Post-test) at *t* = 8.808 (t-value) set a degree of freedom (df: *n* = 119). The effect size of first aid management training on an intentional practice was significantly high (Cohen’s *d* = 2.39) based on the classifications of effect sizes.

### The association between participants’ sociodemographic characteristics profiles and first aid knowledge, attitude, and intention to practice the provision of first aid services to children

To establish the association between participants’ sociodemographic characteristics profiles and First aid knowledge, attitude, and intention to practice the provision of first aid services to children, regression analysis was performed. As shown in Tables 9, 10, and 11 the controlled odds of the first aid training influencing first aid knowledge, attitude, and intention to practice the provision of first aid services to preschool children among preschool teachers were significantly higher (AOR = 2.304; *p* < 0.01; 95%CI: 1.037, 5.939), (AOR = 1.039; *p* < 0.01; 95%CI: 0.658, 2.092) and (AOR = 1.793, *p* < 0.01; 95%CI: 0.985, 3.201) against when they would not be exposed to the training package respectively. Other variables were not significantly associated with the outcome variables as indicated in the table.

**Table 9:** The association between participants’ sociodemographic characteristics profiles and knowledge of first aid services to children

Variable	OR	95% CI		*p*	AOR	95% CI		*p*
		**Lower**	**Upper**			**Lower**	**Upper**	
**Age group**								
18–35 years	0.334	0.127	0.879	0.026	2.993	1.137	7.79	0.094
36–55 years	1							
**Gender**								
Female	0.924	0.277	3.095	0.901	1.388	0.314	6.130	0.666
Male	1							
**Area of residence**								
Urban	0.574	0.233	1.414	0.227	1.231	0.384	3.952	0.727
Rural	1							
**Working Sector**								
Urban	0.599	0.218	1.651	0.322	0.390	0.074	2.046	0.265
Rural	1							
**Educational status**								
Secondary	2.755	1.277	5.957	0.010	2.765	0.074	2.046	0.215
Certificate	0.857	0.090	1.993	0.152	0.424	0.90	1.993	0.277
Diploma	1							
**Teaching experiences**								
Short term	1.595	0.649	3.920	0.309	3.930	0.836	18.417	0.083
Long term	1							
**First Aid Training**								
Before	1							
After	3.421	1.068	6.271	0.001	2.304	1.037	5.939	0.001

**Table 10:** The association between participants’ sociodemographic characteristics profiles and attitude towards first aid services to children

Variable	OR	95% CI		*p*	AOR	95% CI		*p*
		**Lower**	**Upper**			**Lower**	**Upper**	
**Age group**								
18–35 years	0.371	0.157	0.879	0.024	3.210	0.853	12.088	0.085
36–55 years	1							
**Gender**								
Female	2.046	0.533	7.565	0.283	0.983	0.258	3.825	0.991
Male	1							
**Area of residence**								
Urban	1.821	0.754	4.400	1.821	0.726	0.255	2.068	0.549
Rural	1							
**Working Sector**								
Public	0.460	0.184	1.163	0.001	0.503	0.154	1.645	0.256
Private	1							
**Educational status**								
Secondary	1.132	0.614	2.088	0.691	2.372	0.525	10.729	0.262
Certificate	0.424	0.90	1.983	0.277	0.334	0.066	1.692	0.185
Diploma	1							
**Teaching experiences**								
Short term	2.163	0.944	4.957	0.068	0.351	0.086	1.434	0.145
Long term	1							
**First Aid Training**								
Before	1							
After	1.813	0.807	3.083	0.001	1.039	0.658	2.092	0.001

**Table 11:** The association between participants’ sociodemographic characteristics profiles and intention to provide first aid services to children

Variable	OR	95% CI		*p*	AOR	95% CI		*p*
		**Lower**	**Upper**			**Lower**	**Upper**	
**Age group**								
18–35 years	0.769	0.281	2.107	0.610	4.354	0.853	9.011	0.071
36–55 years	1							
**Gender**								
Female	0.603	0.175	2.083	0.042	0.976	0.355	2.527	0.218
Male	1							
**Area of residence**								
Urban	0.594	0.217	1.626	0.310	0.811	0.114	1.689	0.312
Rural	1							
**Working Sector**								
Public	1.456	0.387	5.470	0.578	0.881	0.233	1.702	0.101
Private	1							
**Educational status**								
Secondary	0.662	0.316	1.386	0.274	0.422	0.085	0.845	0.062
Certificate	0.424	0.90	1.993	0.277	0.147	0.029	1.002	0.320
Diploma	1							
**Teaching experiences**								
Short term	0.686	0.239	1.972	0.058	0.416	0.035	1.241	0.213
Long term	1							
**First Aid Training**								
Before	1							
After	2.031	1.177	5.038	0.001	1.793	0.985	3.201	0.001

## Discussion

The response and adherence rate of study participants to the intervention was successfully at 100%, which implies that there was no loss to follow-up from the baseline to the end-line assessments. This study found a positive effect of an intervention on preschool teachers’ knowledge, attitude, and intention to practice provision of the first aid services to preschool pupils. The effect may probably be linked with prescribed materials in the first aid guide that focused more on real-life pediatric injuries scenarios and practical sessions on the best strategies possible to address them. Needless to say, the timing and discussion model of facilitating the training would probably enhance inquiry and participatory learning among pre-school teachers in finding appropriate strategies for managing pediatric injuries in schools, which in turn would influence the effect of the intervention on pre-school teachers’ first aid knowledge, attitude and intention to provide first aid management to pre-school children. Moreover, the inclusion of trained research trainers who also had expertise in emergency care and or teaching would lead the intervention to be more feasible and effective in enhancing preschool teachers’ competencies in providing first aid services to preschool children.

The number of sessions alongside their frequencies was prescribed at an interval that was defined to be enough for pre-school teachers to grasp new knowledge about first aid services and demonstrate it under minimal support from the trained researcher trainers. Thus, findings that may be observed in this study may imply that the delivery of pediatric first aid training among pre-school teachers demonstrates a significant effect on knowledge, attitude, and intention to practice the provision of first aid services to pre-school pupils in Pemba island. Moreover, the intervention seemed to influence interactive communication among pre-school teachers throughout the intervention, which is an essential component in any contemporary educational industry.

In line with the findings of this study, the work of Bandyopadhyay et al*.,* [20] and Hassan et al*.,* Shetie et al*.,* [6] uncovered that a well-designed and implemented first aid health education and training program in the nature of social constructivism grounds improves knowledge and practice of Kindergarten teachers in addressing pediatric emergencies in schools. The similarity may probably be due to the resemblance in the study topic, study population, and or study settings. Moreover, Gharsan et al*.,* [26], suggested that first aid training among preschool teachers promises a healthy survival of children because of the expected competencies demonstrated by teachers after being trained on first aid management to pediatric injuries in schools. Their findings provide insight the same as the current study as multidisciplinary strategies in addressing pediatric injuries among pre-school pupils are very potential than merely leaving the task to health workers.

Méndez [3], Li, Sheng, Zhang, Jiang, and Shen [33] and Li, Jiang, Jin, Qiu, Shen [34] added that it is very important to empower pre-school teachers with first-aid management competencies through well-structured and implemented first-aid training courses grounded in a participatory pedagogical nature because such intervention is the positive predictors of teachers’ first aid responsiveness competencies. Pre-school teachers are the proximal objects to pre-school pupils who need their close attention and timely response to health problems in schools. Empowering preschool teachers with appropriate and specific first aid management strategies may promise the decline of morbidity and mortalities caused by pediatric injuries among children in pre-schools.

Although the findings of this study are limited to the employed self-reported questionnaire that does not measure the actual practices of providing pediatric first aid services to the pre-school pupils, it assessed the intention of the pre-school teachers, which is the primary drive to the practices. The actual practices would have observed trends of events to see how teachers provided pediatric first aid services to document reduced effects of injuries among pre-school pupils within a period. Nevertheless, the study was constrained by including an adequate number of experts such as curriculum developers, clinical experts in emergency and critical care, and educators who would be consulted and provide constructive information during the development and prototyping of pediatric first aid training materials.

Teachers’ Resource Centers require adequate non-human resources such as transport alongside its fairs to the principal investigator and research trainers. However, the current study was constrained to efficient and consistent transport, as it was a rainy season that led to some delays in the timely commencement of some sessions. In implementing pediatric first aid training as in this study, the researcher needs to establish close and frequent consultations with the experts in curricula or program development, meet with educational and health stakeholders to sharpen ideas and pedagogies of the materials, and spare sometimes for prototyping the materials before the main study. The most prominent challenge was to ensure that research trainers adhered to the prescribed pedagogical knowledge and content in the pediatric first aid materials, as there were no fixed cameras to monitor them if they would not integrate other pedagogies, which were not prescribed in the training guide. However, the research team established frequent un-notified visits to the training venues to monitor, support, and emphasize adherence of the trainers to the guide.

## Conclusions

The first aid training for pre-school teachers was valid and feasible in Zanzibar. The end-line findings provide robust evidence that first-aid training holds a potential effect on enhancing knowledge, attitude, and intention to practice the provision of first-aid services to pre-school pupils. The training may be considered an alternative pedagogical approach that might advocate multidisciplinary strategies in ending pediatric injuries among pre-school children in Zanzibar. However, its preparation needs prototyping and frequent and consistent expert consultations to define what and how to organize teaching and learning experiences, define the length of sessions and duration of courses, frequency of sessions, and timing for evaluating it. This study recommends regular professional development training programs on managing pediatric injuries in schools among preschool teachers in Zanzibar to ensure that children’s health is secure enough for their healthy adulthood and future investment.  

## Data Availability

The datasets used and/or analyzed during the current study are available from the corresponding author on reasonable request via wcle87@gmail.com or walter.millanzi@udom.ac.tz or will be available at the institutional repository address http://repository.udom.ac.tz at the time of publication.
